# CD8 T-Cell Responses in Incident and Prevalent Human Papillomavirus Types 16 and 18 Infections

**DOI:** 10.5402/2012/854237

**Published:** 2012-03-04

**Authors:** Hannah N. Coleman, Anna-Barbara Moscicki, Sepideh N. Farhat, Sushil K. Gupta, Xuelian Wang, Mayumi Nakagawa

**Affiliations:** ^1^Department of Pathology, College of Medicine, University of Arkansas for Medical Sciences, 4301 West Markham Street, Slot 502, Little Rock, AR 72205, USA; ^2^Department of Pediatrics, College of Medicine, University of California, San Francisco, 3333 California Street, San Francisco, CA 94143, USA; ^3^Department of Microbiology and Parasitology, College of Basic Medical Sciences, China Medical University, Shenyang 110001, China

## Abstract

CD8 T-cell responses were examined in subjects with incident (new following negative visits) or prevalent (lasting ≥ 4 months) human papillomavirus type 16 (HPV16) or human papillomavirus (HPV18) infection. The groups were chosen from a cohort of women being followed every 4 months with cervical cytology and HPV-DNA testing. Enzyme-linked immunospot (ELISPOT) assay was performed at enrollment (time zero) and one year later. At time zero, 1 (6%) of 17 subjects with incident HPV 16/18 infections had positive ELISPOT results which increased to 6 (35%) at one year. For the subjects with prevalent HPV 16/18 infections, the ELISPOT results were similar at time zero (2 (15%) of 15 subjects positive) and at one year (3 (20%)). While all of the 11 women with prevalent HPV16 infection showed clearance one year later, unexpectedly only 1 (25%) of 4 women with prevalent HPV18 infection demonstrated clearance one year later (*P* = .009).

## 1. Introduction

A majority of human papillomavirus (HPV) infections is cleared within a few years [1, 2]. However, persistent infections of the cervix by high-risk HPV types are associated with the development of cervical cancer [3, 4]. In patients infected with human immunodeficiency virus (HIV), the nature of T-cell immune responses in primary infections was shown to be important in viral control and were found to be quite different from that in chronic infections [5]. In order to compare the nature of immune responses between short-term and long-lasting HPV infections, we examined T-cell responses in subjects with incident (new infection detected following HPV negative visits) and prevalent (lasting ≥ 4 months) human papillomavirus type 16 (HPV16) or human papillomavirus type 18 (HPV18) infection. The subjects were recruited from a long-term study of the natural history of HPV infection.

## 2. Methods

### 2.1. Subjects

**Figure 1:**
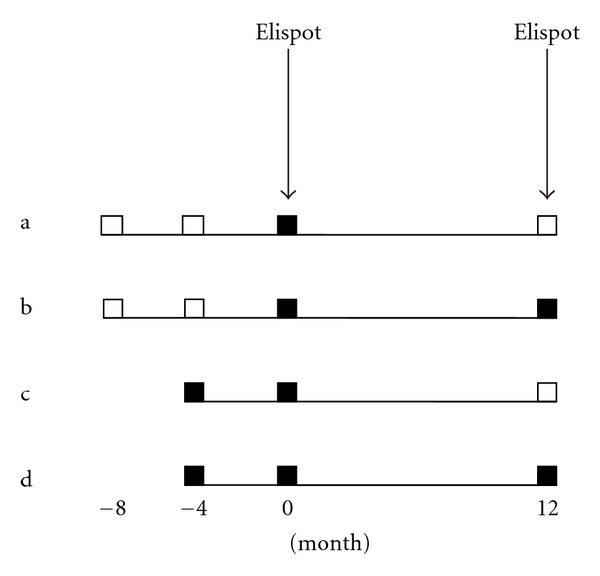
Natural history of HPV16 or HPV18 infection and time points at which ELISPOT assays were performed. (a) Schematic representation of an incident HPV16 or HPV18 infection which cleared one year later. (b) Schematic representation of an incident HPV16 or HPV18 infection which persisted one year later. (c) Schematic representation of a prevalent HPV16 or HPV18 infection which lasted at least four months at the time of study entry and which cleared one year later. (d) Schematic representation of a prevalent HPV16 or HPV18 infection which lasted at least four months at the time of study entry and which persisted one year later. “■”: a visit at which HPV16 or HPV18 DNA was detected. “□”: a visit at which HPV-DNA testing result was negative for HPV16 and HPV18.

The parent study enrolled young women aged 13–21 years with sexual experience of less than 5 years [6]. Interview, cervical cytology, and HPV-DNA testing using cervical lavage specimen were performed every 4 months. For the current study, two groups were chosen: (1) women with an incident HPV16 or HPV18 infection (undetectable HPV-DNA results for two consecutive visits followed by a positive one) and (2) women with a prevalent HPV16 or 18 infection (those with at least two consecutive visits positive for HPV16 or HPV18) (Figure 1). The HPV-DNA testing results one year later were then used to determine whether the incident or prevalent infection was still detectable. In addition, 20 subjects who never had HPV16 and HPV18 detected were enrolled as negative controls. Approvals were obtained from the Committee on Human Research of the University of California at San Francisco and from the Institutional Review Board of the University of Arkansas for Medical Sciences, and informed consent was obtained from each subject. 

### 2.2. HPV-DNA Testing

 The cervicovaginal lavage samples were tested using the PGMY09/11 primer system as previously described [6]. The PCR amplified product was tested with a reverse line blot assay (Roche Molecular Diagnostics, Inc., Alameda, CA) for the presence of a positive **β**-globin signal (indicating sample adequacy) and for the following HPV types: 6, 11, 16, 18, 26, 31, 33, 35, 39, 40, 42, 45, 51, 52, 53, 54, 55, 56, 57, 58, 59, 66, 68, 73, 82, 83, and 84.

### 2.3. Cloning and Expression of HPV18 E6 and E7 Proteins

 Recombinant vaccinia viruses expressing HPV18 E6 and E7 separately, used to establish CD8 T-cell lines, were constructed in the same manner as the HPV16 E6-vac and E7-vac [7]. The E6 and E7 genes were PCR-amplified from a plasmid containing HPV18 [8] using primers designed to insert Kpn I and Sal I restriction enzyme sites. The amplified and digested DNA fragments were ligated into pSC65 [9]. The resultant plasmid transfer vectors contained inserted open reading frames regulated by a strong synthetic early/late vaccinia promoter and the *lacZ* gene regulated by the P7.5 early/late vaccinia virus promoter flanked by vaccinia virus thymidine kinase gene. The E6 and E7 genes from the plasmid transfer vectors were sequenced, and the results matched with the sequences from the GenBank. Cells were infected with the WR wild-type vaccinia virus strain and transfected with the plasmid transfer vector containing the E6 or E7 gene. The recombinants (HPV18 E6-vac E7-vac, resp.) were isolated by TK selection, and plaques were purified. CV-1 cells were infected with HPV18 E6-vac or E7-vac, and cell lysates were resolved on a SDS-PAGE gel, which was transferred to a Zeta-Probe blotting membrane (Bio-Rad Laboratories, Hercules, CA). The expression of the HPV18 E6 or E7 protein was confirmed by Western blotting performed using HPV 16/18 E6-specific monoclonal antibody (C1P5, Chemicon International, Temecula, CA) or HPV18 E7-specific antibody (718-15, Abcam Inc., Cambridge, MA).

### 2.4. Enzyme-Linked Immunospot (ELISPOT) Assay

 Blood samples to establish HPV16- or HPV18-specific CD8 T-cell lines and to perform interferon-*γ* ELISPOT assay [10, 11] were drawn as soon as incident or prevalent infection was known. A second blood draw was performed one year later. Peripheral blood mononuclear cells (separated magnetically into CD14-positive cells and CD14-negative cells) were cryopreserved, and both samples were analyzed simultaneously. The HPV16-based ELISPOT assay for E6 and E7 proteins was performed for subjects with HPV16 infection as previously described [11], and the same was done for HPV18. The E6 protein was tested using 10 pools of 3 overlapping synthetic 15-mer peptides for HPV16 [11] and for HPV18. Six pools of three 15-mer synthetic peptides were used to cover the HPV16 E7 protein, while six pools and an additional peptide, HPV18 E7 (91–105), were tested for the HPV18 E7 protein. CD8 T-cell line was established, and ELISPOT assay was performed once for the 20 negative control subjects: 10 subjects were tested with the HPV16-based assay, and the other 10 subjects were tested with the HPV18-based assay.

### 2.5. Statistical Analysis

Statistical analyses were performed using GraphPad Software (GraphPad Software Inc., La Jolla, CA). Fisher's exact test (two tailed) was used to make comparisons between 2 groups, and unpaired *t*-test was used to compare the duration of persistence prior to entry between the HPV16 and HPV18 prevalent groups. A *P* value of less than  .05 was considered to be significant.

## 3. Results

The mean age of subjects at enrollment for this substudy was 23.4 years. The first blood samples were drawn within a mean of 77 ± 40 days after the incident and prevalent infections were known. At time zero, 1 (6%) of 17 subjects with incident HPV 16/18 infections had positive ELISPOT results which increased to 6 (35%) at 1 year (*P* = .09). After one year, 12 (71%) of 17 subjects with an incident HPV 16/18 infections showed clearance (Table 1). However, there was no difference in ELISPOT results between the subjects whose HPV 16/18 infections cleared (4 of 12 or 33%) versus persisted (2 of 5 or 40%, *P* = 1) at one year point.

For the subjects with prevalent infection, the mean duration of persistence prior to enrollment in this substudy was  513 ± 139  days for the HPV16 group and 916 ± 208 days for the HPV18 group (*P* = .1). At time zero, 2 (15%) of 15 subjects with prevalent HPV 16/18 infections had positive ELISPOT results while 3 (20%) of them did at 1 year (*P* = 1). Three (25%) of 12 subjects whose prevalent HPV 16/18 infections cleared had positive ELISPOT results while none (0%) of 3 subjects whose infections persisted (*P* = 1) at one year. Interestingly, all of the 11 women with prevalent HPV16 infection showed clearance one year later. In comparison, only 1 (25%) of 4 women with prevalent HPV18 infection demonstrated clearance one year later (*P* = .009) (Table 1). The rate of positive CD8 T-cell responses was 27% (3 of 11) among those with prevalent HPV16 infections and 0% (none of 4) among those with prevalent HPV18 infections one year later (*P* = .5).

None of the negative control subjects had any positive ELISPOT results. There was a significant difference between the ELISPOT results of the negative control group and the ELISPOT results at one year after the incident HPV 16/18 infections (6 (35%) of 17 women had positive ELISPOT results (*P* = .005)).

## 4. Discussion

The CD8 T-cell immune responses mount more frequently after incident HPV 16/18 infections (6% to 35%) compared to prevalent HPV 16/18 infections (15% to 20%). However, only a statistical trend was demonstrated due to small number of subjects. These results are quite different from those examining HIV-positive subjects in which robust systemic CD8 T-cell responses were demonstrated during primary and chronic infections although their roles in controlling HIV infections were distinct [5]. The presence of robust systemic immunity in HIV compared to HPV infections may be due to the fact that HIV causes systemic infections while HPV causes localized infections such as in cervix.

 A surprising finding was that persistence one year later was more common for HPV18 compared to HPV16 among those women who were found to have prevalent infection at enrollment. There seems to be an immunological basis for the difference (27% for HPV16 versus 0% for HPV18 at 1 year), but it was not statistically significant unlike the findings in other studies for which we performed the same assay [10, 11]. This was likely due to the small number of subjects in this study. The finding that the prevalent HPV18 infection persisted more often than the prevalent HPV16 infection was unexpected given that other investigators have reported HPV16 to be the type that most commonly persisted [12, 13] although the time periods observed were much longer in these studies (5 years and 5–7 years, resp.). Although the duration of persistence for the HPV18 prevalent group was longer than that for the HPV16 prevalent group, this difference was not statistically significant. While a longer duration of persistence is thought to lead to further persistence [14], this difference alone did not seem to explain the significant difference in the HPV persistence 1 year later between the HPV16 and HPV18 prevalent groups in this study. HPV18 infection is associated with glandular lesions that are often difficult to detect by cytology [15]. Therefore, it is tempting to speculate that HPV18 infection may take a different course compared to other high-risk HPV types. Indeed, 4 of 9 cases of cervical cancer diagnosed at the 2nd observations were associated with HPV18 in the study reported by Schiffman and colleagues [13]. Findings from another study suggested that HPV18 may be more oncopotent in some population than HPV16 although the latter was more prevalent [16]. The weakness of this study was the small number of subjects enrolled; however, it is extremely difficult to accumulate sufficient number of subjects with a well-characterized history of HPV16 or HPV18 infection. Nevertheless, it would be important to corroborate the findings of this study.

## 5. Conclusion

A trend indicating the CD8 T-cell immune responses mount more frequently after incident HPV 16/18 infections compared to prevalent HPV 16/18 infections was described, but an analysis of a larger number of subjects is warranted. Unexpectedly, HPV18 was detected significantly more frequently than HPV16 one year following prevalent infection. Whether this was due to less CD8 T-cell responses mounted against HPV18 compared to HPV16 was not clear.

## Figures and Tables

**Table 1:** Course of HPV infections.

HPV Type	HPV16		HPV18	
One Year Later	Cleared, no (%)	Still Present, no (%)	Cleared, no (%)	Still Present, no (%)

Incident	9 (75)	3 (25)	3 (60)	2 (40)
Prevalent	11 (100)	0 (0)	1 (25)	3 (75)
